# Impact of meteorological parameters and population density on variants of SARS-CoV-2 and outcome of COVID-19 pandemic in Japan

**DOI:** 10.1017/S095026882100100X

**Published:** 2021-04-28

**Authors:** Nadim Sharif, Shamsun Nahar Ahmed, Rubayet Rayhan Opu, Muktasid Ud Daullah, Shahriar Khan, Ali Azam Talukder, Shoko Okitsu, Hiroshi Ushijima, Ming Zhang, Shuvra Kanti Dey

**Affiliations:** 1 Department of Microbiology, Jahangirnagar University, Savar, Dhaka 1342, Bangladesh; 2 Division of Microbiology, Department of Pathology and Microbiology, Nihon University, Tokyo, Japan; 3 Department of Epidemiology and Biostatistics, College of Public Health, University of Georgia, Athens, GA, USA

**Keywords:** Cluster mutations, COVID-19, Japan, population density, variants, weather

## Abstract

Although vaccines have become available, emergence and rapid transmission of new variants have added new paradigm in the coronavirus disease-2019 (COVID-19) pandemic. Weather, population and host immunity have been detected as the regulatory elements of COVID-19. This study aims to investigate the effects of weather, population and host factors on the outcome of COVID-19 and mutation frequency in Japan. Data were collected during January 2020 to February 2021. About 92% isolates were form GR clades. Variants 501Y.V1 (53%) and 452R.V1 (24%) were most prevalent in Japan. The strongest correlation was detected between fatalities and population density (*r*_s_ = 0.81) followed by total population (*r*_s_ = 0.72). Relative humidity had the highest correlation (*r*_s_ = −0.71) with the case fatality rate. Cluster mutations namely N501Y (45%), E484K (30%), N439K (16%), K417N (6%) and T478I (3%) at spike protein have increased during January to February 2021. Above 90% fatality was detected in patients aged >60 years. The ratio of male to female patients of COVID-19 was 1.35:1. This study will help to understand the seasonality of COVID-19 and impact of weather on the outcome which will add knowledge to reduce the health burden of COVID-19 by the international organisations and policy makers.

## Introduction

The ongoing pandemic namely, coronavirus disease-2019 (COVID-19) has been triggered by the infection of a novel species of coronavirus (named 2019-novel coronavirus, 2019-nCoV) called severe acute respiratory syndrome coronavirus-2 (SARS-CoV-2) of genera *Betacoronavirus*, family Coronaviridae [1–3]. Human coronavirus 229E and OC43 were the first members of Coronaviridae detected in patients with the symptoms of respiratory system infection and common cold during early 1960s [4]. Other members of the same genus *Coronavirus* namely, SARS-CoV, HCoV NL63, HKU1 and MERS-CoV had caused local outbreaks with human respiratory system infections in 2003, 2004, 2005 and 2012, respectively [1–5]. Among coronaviruses, only SARS-CoV-2 has caused a severe and global pandemic [1–5].

SARS-CoV-2 is a positive sense, non-segmented single-stranded RNA (ssRNA) virus with a genome of ~30 000 bases [1, 6]. The genome is consisted of a 5′ cap structure, 10 open-reading frames (ORFs) namely, 1a, 1b, 3a, 3b, 6, 7a, 7b, 8a, 8b and 9b along with a 3′ poly (A) tail [7–9]. First two ORFs from 5′ end namely ORF-1ab comprise of ~20 000 bases and encode for non-structural proteins (nsps) (replicase proteins). About 16 nsps (nsp1–nsp16) and four major structural proteins – spike (S), envelope (E), membrane (M) and nucleocapsid (N) have been identified and characterised [7–11]. Major structural proteins of SARS-CoV-2 are encoded by the later ORFs of 3′ end (~10 000 bases) [1, 2, 7–11].

Various clinical manifestations have been reported from patients infected with COVID-19 [1, 5]. The clinical manifestations can be characterised into serve, mild and asymptomatic according to the duration and health outcome [1, 5, 12]. About 65% patients are asymptomatic [12–14]. Among the symptomatic patients about 80–85% develop mild symptoms [1, 12–16]. In mild cases, fever, cough and sore throat are the most common clinical features followed by chill, feelings of shaking, loss of taste or smell, headache, rash and muscle pain. Asymptomatic patients and patients with mild symptoms have a good recovery rate [1, 5, 15, 16]. Clinical manifestations including acute pneumonia, acute respiratory syndrome, kidney failure, difficulty in breathing and failure of multiple organs have been reported in severe cases [12–16].

The first confirmed case of COVID-19 was reported from Wuhan, China in December 2019 [1]. As of 15 February 2021, about 110 million cases and 2.5 million fatalities of COVID-19 have been confirmed from more than 221 countries and territories globally [17, 18]. A sudden increase of COVID-19 cases, fatalities and transmission has been detected from October 2020 [17]. The first COVID-19 case was confirmed in January 2020 in Japan [19, 20]. As of 21 February 2021, about 400 000 cases and 7000 casualties associated with COVID-19 have been detected in Japan [17–21]. About 80% cases and fatalities have been detected after 15 October 2020 in Japan. The case fatality rate has remained 18 persons per thousand people during the first and second waves in Japan [17, 20]. Among 47 prefectures, Tokyo (~100 000 cases, 25%) had the highest number of cases followed by Osaka (~40 000 cases, 10%), Kanagawa (39 000 cases, 10%), Saitama (25 000 cases, 6%), Chiba (22 000 cases, 6%), Aichi (17 000 cases, 4%), Hokkaido (16 000 cases, 4%) and Hyogo (15 000 cases, 4%), respectively. Population density of Japan is 900 persons/mi^2^ with a collective population of about 126 000 000. As of 15 February 2021, a total of ~7 800 000 tests have been recorded with 5.3% positive incidence [17, 18, 20].

The spread of COVID-19 is significantly affected by weather [21]. Meteorological parameters including relative humidity (RH), ultraviolet (UV) intensity, wind velocity, ambient temperature, rain fall and snowfall have contributed significantly to shape the wave of COVID-19 pandemic [22–25]. Weather parameters regulated the viability, and transmission of COVID-19 [21–24]. Furthermore, total population, density of population, duration of lockdown, tendency of common people to maintain social distance and COVID-19-related health practices, movement frequency, transportation of infected persons across borders, gatherings during sports, social and religious events etc. have contributed significantly to the spread of COVID-19 [22–28].

Mutation at receptor binding sites (RBDs) of spike protein is mainly involved in the origin of recent variants of SARS-CoV-2 [29–31]. Deletion, indels and cluster of substitution point mutations at RBDs influence the origination of escape variants with a high growth rate. Numerous parameters of host and weather influence the mutational events [30–32]. Inside host body, the immune system, presence of coinfection and antiviral drug pressure are shaping mutations in coronavirus genome. Besides, parameters of weather specifically UV radiation, high ambient temperature, RH and snowfall have regulatory roles in evolving mutant viruses [22–25].

The principal objective of this study is to investigate the impact of regulatory factors specially, weather on mutation frequency in SARS-CoV-2 in Japan. Other objectives of this study are to specify the relationship between mutations at RBDs and host factors, and to determine the impact of weather on the outcome of COVID-19 pandemic in Japan. We also analysed the impact of population density, social gatherings and lockdown on the incidence and casualties in Japan. This study will create a better understanding of the effects of various regulatory factors on mutations of SARS-CoV-2 and outcome of COVID-19 in Japan.

## Materials and methods

### Study regions and time period

This study determined the impact of weather on COVID-19 in Japan. Eight regions in Japan namely, Hokkaido (44°N to 143°E), Tohoku (35°82′N to 139°57′E), Kanto (36°45′N to 139°69′E), Chubu (35°1′N to 136°53′E), Kansai (34°6′N to 135°5′E), Chugoku (35°8′N to 104°1′E), Shikoku (33°7′N to 133°6′E) and Kyushu (32°5′N to 130°8′E) with 47 prefectures were included in this study. This study was conducted during February 2020 to February 2021.

### Study data collection and data availability

The data regarding the COVID-19 pandemic were retrieved by using an unbiased approach from official databases of Japan Government (https://www.mhlw.go.jp/stf/seisakunitsuite/bunya/0000164708_00079.html), COVID-19 information and resources (https://corona.go.jp/en/) and coronavirus situation report in Japan (https://toyokeizai.net/sp/visual/tko/covid19/en.html). Collected data were cross-matched by analysing the COVID-19 data form Word Health Organization (https://www.who.int/emergencies/diseases/novel-coronavirus-2019), Worldometers (www.worldometers.info/coronavirus/) and COVID-19 dashboard by Johns Hopkins University (https://coronavirus.jhu.edu/map.html).

Weather data including maximum temperature (°C), average temperature (°C), minimum temperature (°C), UV index, snowfall, precipitation, wind velocity (km/h), rain fall (mm) and RH (%) were retrieved from Japan Meteorological Agency (https://www.jma.go.jp/jma/indexe.html) and weather underground (https://www.wunderground.com/global/JP.html). The collected meteorological data were cross-checked with the data from AccuWeather (www.accuweather.com), meteoblue (www.meteoblue.com) and Yahoo Japan (https://weather.yahoo.co.jp/weather/) in this study. SARS-CoV-2 whole genome analysis was conducted using reference genome sequences from the website of GISAID (https://www.gisaid.org/) [33]. Data on the occurrence of gatherings, social and religious events, sports events and lockdown were collected from the government of Japan website (https://www.japan.go.jp/) and Japan government news (https://www.japan.go.jp/kizuna/).

### Whole genome and mutational analysis of SARS-CoV-2 variants

The whole genome sequences of SARS-CoV-2 variants in Japan were analysed using Chromas 2.6.5 (Technelysium, Helensvale, Australia). Sequence homology of the selected whole genomes was deduced by using the nucleotide BLASTn program of NCBI (https://blast.ncbi.nlm.nih.gov/Blast.cgi). Using the reference strains (NC_045512/Wuhan-Hu-1) and whole genome sequences of Japanese SARS-CoV-2 isolates multiple sequence alignment was performed by operating ClustalW Multiple Alignment algorithm in BioEdit 7.2.6 software. Clades were defined according to the presence of specific markers following the GISAID system [33]. Nucleotide and peptide sequences of whole genome of the selected isolates were analysed by using MEGA X to determine deletion, indels and substitution point mutations [9, 22].

### Statistical analysis

All data were sorted and analysed using unbiased statistical approaches. Appropriate correlation analysis was performed to determine the association between two variables by using a monotonic function of Spearman's rank correlation coefficient (*r*_s_) [22]. The Spearman's rank correlation coefficients (*r*_s_) were calculated between weather factors and cases as well as weather factors and casualties [22, 25]. Furthermore, associations between host factors and mutation frequency, weather parameters and mutational events of SARS-CoV-2 were also determined by using the Spearman's rank correlation coefficient (*r*_s_). The below equation is used to calculate the coefficient in this study:
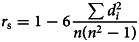
Here, ‘*n*’ represents the number of observations, ‘*d_i_*’ represents the difference between the ranks of observations and *r*_s_ represents the Spearman's correlation coefficient.

## Results

### Temporal and spatial distribution of COVID-19 cases and fatalities

**Fig. 1.**
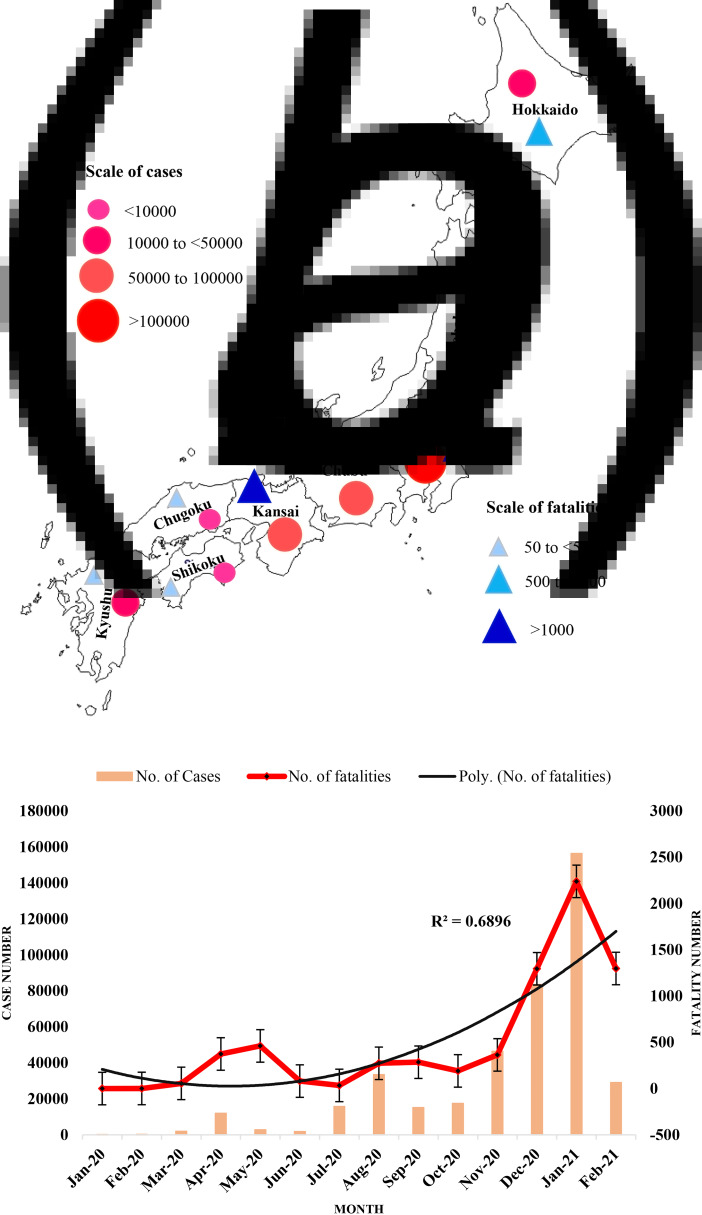
(a) Map of spatial distribution of COVID-19 cases and fatalities in eight regions in Japan during January 2020 to February 2021 and (b) monthly distribution of total cases and fatalities of COVID-19 in Japan.

A sharp increase in both cases and fatalities of COVID-19 has been detected after October 2020 in Japan. About 75% of cases and fatalities have occurred during October 2020 to February 2021 (Fig. 1a and b). Although the cases and casualties have increased, the case fatality rate has remained constant near 1.8 in Japan. All of the 47 prefectures in eight regions have been hit by the second wave of COVID-19. Kanto is burdened with the highest number of cases (215 000), whereas Kansai has the most number of fatalities (about 1800) (Table 1). The number of cases in Kanto is followed by Kansai (~77 000), Chubu (~57 000), Kyushu (~34 000) and Hokkaido (~18 000), respectively (Table 1). Twenty prefectures burdened with the most number of cases and fatalities have been studied. The highest number of cases was detected in Tokyo (~100 000) followed by Osaka (~44 000) and other prefectures. The highest number of fatality was found in Osaka (~1000), whereas Hokkaido had the highest case fatality rate (Table 2).

**Table 1.** Distribution of COVID-19 cases, fatalities, case fatality rate and death rate in eight different regions in Japan

Regions	No. of cases	No. of fatalities	Case fatality rate per 10 000 cases	Death rate per 100 000 persons
Kanto	215 000	1726	80	4
Kansai	77 000	1740	226	8
Chubu	57 100	781	137	4
Kyushu	33 500	496	148	3
Hokkaido	17 514	550	314	10
Chugoku	8170	141	173	2
Tohoku	3400	133	391	1
Shikoku	2700	55	204	1

**Table 2.** Trends of total COVID-19 cases, fatalities and case fatality rate in the top 20 prefectures in Japan

Prefecture	No. of total cases	No. of total fatalities	Case fatality rate per 10 000 cases
Tokyo	100 302	891	89
Osaka	43 901	930	212
Kanagawa	41 035	482	117
Saitama	25 384	359	141
Aichi	24 126	402	167
Chiba	22 518	254	133
Hokkaido	17 514	607	347
Hoyogo	16 580	412	248
Fukuoka	16 247	192	118
Kyoto	8510	122	143
Hiroshima	4828	92	191
Shizuoka	4608	77	167
Tochigi	3775	48	127
Miyagi	3457	22	64
Okayama	2345	23	98
Nagano	2290	35	153
Shiga	2132	31	145
Fukushima	1735	45	259
Nagasaki	1530	28	183
Yamagata	510	13	255

### Analysis of factors of weather

**Fig. 2.**
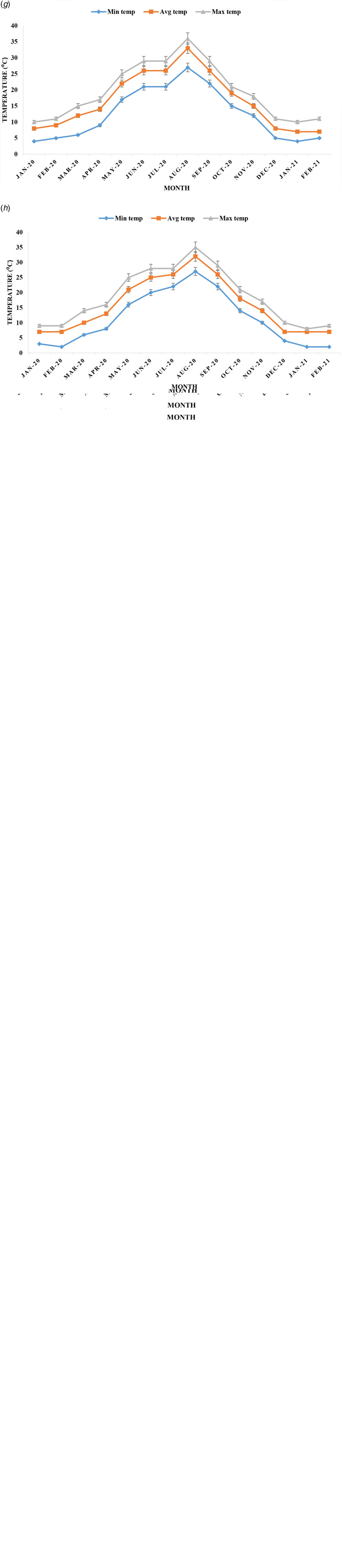
Monthly distribution of minimum temperature (min temp), average temperature (avg temp) and maximum temperature (max temp) in (a) Kanto; (b) Kansai; (c) Chubu; (d) Kyushu; (e) Hokkaido; (f) Chugoku; (g) Tohoku and (h) Shikoku during January 2020 to February 2021 in Japan.

Amid parameters of weather, temperature, UV ray intensity, RH, rainfall, snowfall, wind speed, atmospheric pressure and sun hours were analysed for their role in COVID-19. Three temperatures were documented during January 2020 to February 2021. The mean of maximum temperature was about 15 °C, and ranged from −3 to 41 °C in eight regions in Japan. The mean of average temperature was 13 °C and minimum temperature was 8 °C in Japan during the last 14 months of COVID-19 pandemic (Fig. 2a–h). The mean values of minimum, maximum and average temperature varied ±6 °C in eight regions. The lowest temperature was detected in Hokkaido (−16°C) in February 2020. A peak of three temperatures in eight regions was confined in August 2020 in Japan. Temperature started to decrease and both cases and fatalities of COVID-19 started to increase from September 2020.

**Fig. 3.**
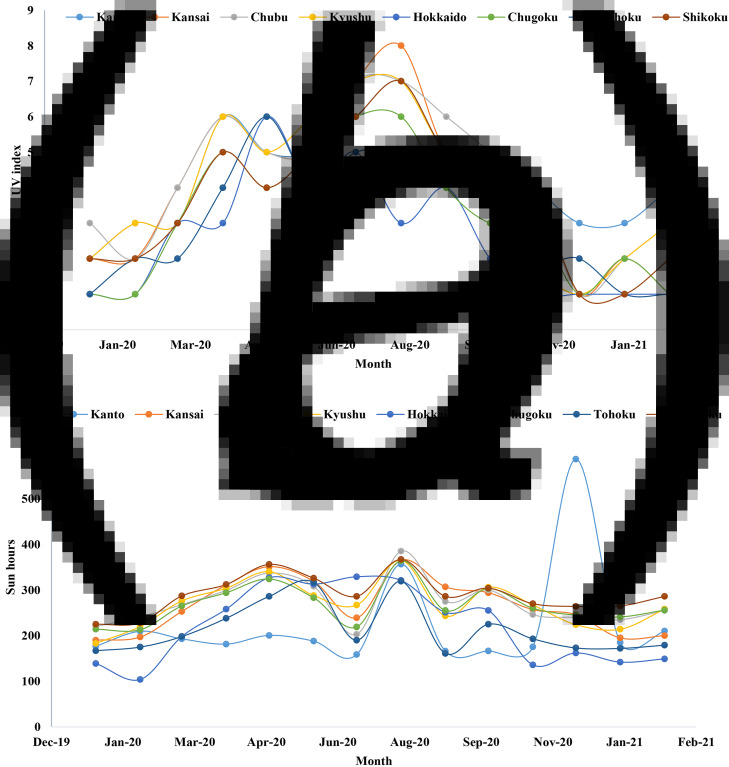
Distribution of average (a) UV index and (b) sun hours per month in Japan.

Sun hours or the amount of day light duration and UV ray intensity are important weather factors that regulate the spread and mutations of SARS-CoV-2. The lowest average UV intensity was 1 in Hokkaido during January 2021. The highest average UV intensity was 9 in Kansai during July 2020 (Fig. 3a). The average value of UV intensity ranged from 1 to 9 during the COVID-19 pandemic. One peak of average UV intensity was confined during April 2020 and another one during August 2020. After August 2020, UV index declined while the cases and fatalities increased. The sun hours ranged from 95 to 600 h per month. A peak of the sun hours was also detected in August 2020 (Fig. 3b).

**Fig. 4.**
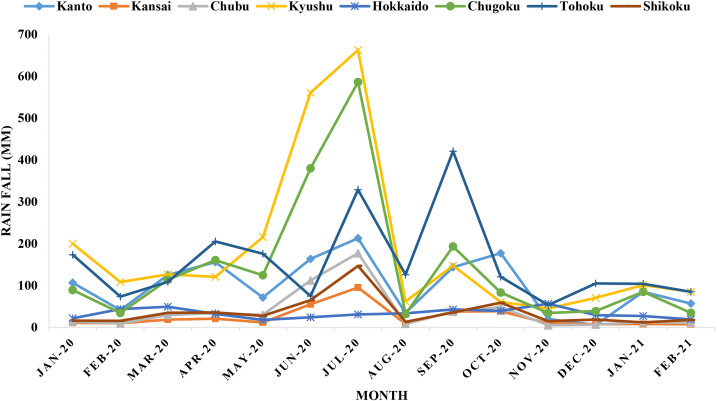
Monthly trends of average rainfall in Japan during the COVID-19 pandemic.

The amount of rainfall and snowfall were also analysed in this study. The highest rainfall was detected in Kyushu (690 mm) in July 2020 followed by Chugoku (580 mm) and Tohoku (420 mm) (Fig. 4). Snowfall was not persistent in eight regions. Only seasonal snowfall was recorded in most of the regions in Japan. The highest rainfall was detected in Hokkaido during February 2020 and 2021.

**Fig. 5.**
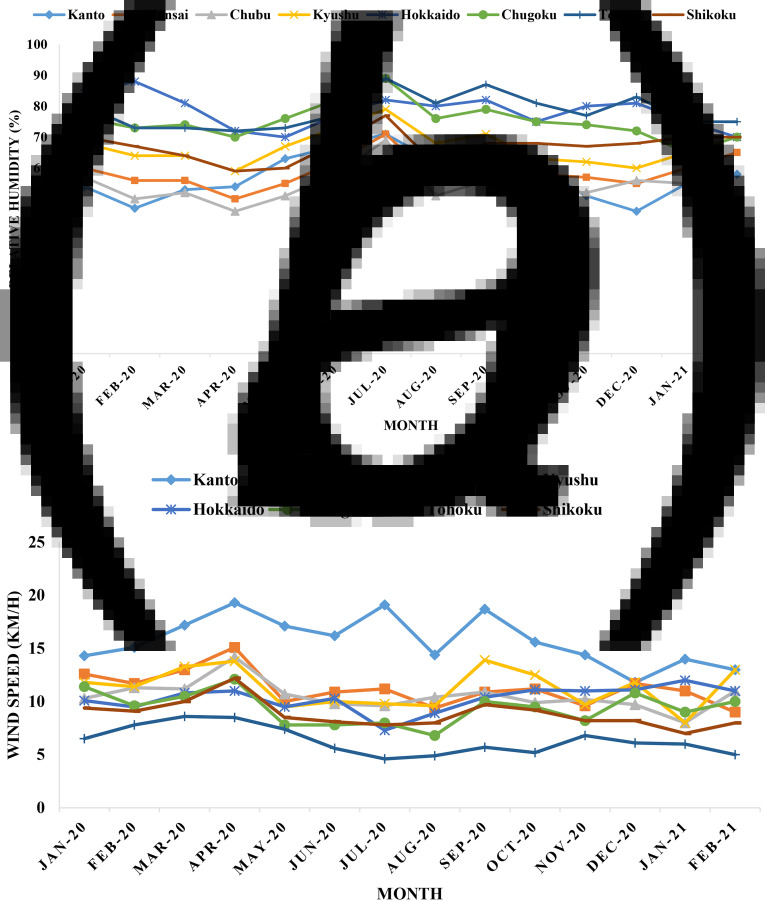
Distribution of average (a) RH and (b) wind speed in eight regions in Japan.

RH, wind speed and atmospheric pressure are also significant factors of weather related to COVID-19. Average RH ranged from 45% to 90% during January 2020 to February 2021 in Japan (Fig. 5a and b). The highest value of RH was recorded in Chugoku (90%) and Tohoku (90%) in July 2020, followed by Hokkaido (85%) in February 2020. A peak of average RH for eight regions in Japan was detected in July 2020. The highest average RH was detected in Hokkaido (80%) followed by Chugoku (78%) and Tohoku (77%), respectively. Atmospheric pressure varied around 1 atm during the pandemic in Japan.

Velocity of wind was recorded and analysed. The average wind speed per month was detected between 4 and 20 km/h in Japan. The highest average wind speed was detected in Kanto (16 km/h) followed by Kyushu (13 km/h) and Kansai (12 km/h), respectively (Fig. 5a and b). The growth rate of cases in Kansai was the highest. The lowest average wind speed was detected in Tohoku during the COVID-19 pandemic.

The total population and population density of eight regions in japan were also analysed. Kanto (43.4 million) had the highest number of total population followed by Kansai (22.6 million), Chubu (21.4 million), Kyushu (14.5 million), Tohoku (8.9 million), Chugoku (7.3 million), Hokkaido (5.4 million) and Shikoku (3.8 million), respectively. Kanto (1300 persons/km^2^) was the most densely populated region followed by Kansai (690 persons/km^2^), Chubu (320 persons/km^2^), Kyushu (307 persons/km^2^), Chugoku (240 persons/km^2^), Shikoku (204 persons/km^2^), Tohoku (130 persons/km^2^) and Hokkaido (63 persons/km^2^), respectively. Tokyo is the capital and centre of most of the international flights and transportation in Japan. The health burden of COVID-19 was also highest in Tokyo and Kanto.

### Correlation analysis between weather and COVID-19 pandemic in Japan

The impact of factors of weather on the outcome of COVID-19 was determined by Spearman's correlation analysis. Twelve factors of weather were considered on three time frames, namely on the day of the incidence, 7 days after and 14 days after the incidence in this study. Four parameters of COVID-19 namely, cases, fatalities, case fatality rate and growth rate were defined as the outcome of the pandemic. The Spearman's rank correlation was calculated between each factor of weather and outcome of COVID-19. Minimum temperature (min temp) on the day of the cases had the highest correlation with the cases (*r*_s_ = −0.55) followed by average temperature (avg temp) on the day (*r*_s_ = −0.51), sun hours on the day (*r*_s_ = −0.49) and RH on the day (*r*_s_ = −0.47), respectively. Both the total population (*r*_s_ = 0.59) and population density (*r*_s_ = 0.47) were strongly correlated with the increase of cases (Table 3). The cumulative fatalities had the highest correlation with population density (*r*_s_ = 0.81) followed by total population (*r*_s_ = 0.72), RH (*r*_s_ = −0.59) and minimum temperature (*r*_s_ = −0.54) on the day, respectively. The highest correlation of case fatality rate was detected with RH (*r*_s_ = −0.71) and sun hours (*r*_s_ = −0.54) on the day. The growth rate of cases had also significant association with population density (*r*_s_ = 0.51) and total population (*r*_s_ = 0.43) (Table 3)

**Table 3.** Spearman's correlation analysis of parameters of weather and outcome of COVID-19 pandemic in Japan

Environmental factors	COVID-19 cases	COVID-19 fatalities	Case fatality rate	Growth rate
Max temp on the day	−0.12	−0.21*	−0.01	−0.001
Max temp 7 days ago	−0.01	−0.12*	−0.001	−0.04
Max temp 14 days ago	−0.14	−0.09	−0.2*	−0.008
Avg temp on the day	−0.51***	−0.27**	−0.14	−0.12
Avg temp 7 days ago	−0.32*	−0.19	−0.19*	−0.14
Avg temp 14 days ago	−0.24	−0.17	−0.21	−0.28**
Min temp on the day	−0.55**	−0.54*	−0.05	−0.07
Min temp 7 days ago	−0.14	−0.38*	−0.009	−0.02
Min temp 14 days ago	−0.21	−0.06	−0.02*	−0.005
UV index on the day	−0.42*	−0.22**	−0.34*	–
UV index 7 days ago	−0.31*	−0.08	−0.39*	−0.006
UV index 14 days ago	−0.11	−0.001	−0.41	−0.002
Sun hours on the day	−0.49**	−0.31	−0.54	−0.41
Sun hours 7 days ago	−0.35	−0.30	−0.39	−0.31*
Sun hours 14 days ago	−0.33	−0.09	−0.14	−0.12
RH on the day	−0.47	−0.59*	−0.71	−0.31
RH 7 days ago	−0.40	−0.46	−0.42*	−0.18
RH 14 days ago	−0.41	−0.41**	−0.37	−0.004
Rainfall on the day	−0.02*	−0.27	−0.04*	−0.0004
Rainfall 7 days ago	0.003	−0.01	0.31	0.002
Rainfall 14 days ago	−0.0002	−0.0001	−0.21	−0.34
Wind speed on the day	0.27*	0.12**	0.39*	0.14*
Wind speed 7 days ago	0.14*	0.01*	0.27*	0.10*
Wind speed 14 days ago	0.13	0.0009	0.19	0.09
Total population	0.59**	0.72*	0.48**	0.43**
Population density	0.47*	0.81*	0.34*	0.51*

**, * stand for 1% and 5% levels of significance.

First, the correlation between maximum temperature and the number of total cases in each city was evaluated. Similarly, the correlation between minimum temperature and total cases, average temperature and total cases were determined. Besides, correlations among three temperatures with total fatalities in each city were also determined. The average temperature on the day of the cases had the highest correlation (*r*_s_ = −0.675), followed by average temperature 7 days ago (*r*_s_ = −0.547), maximum temperature on the day (*r*_s_ = −0.512) and minimum temperature on the day (*r*_s_ = −0.486). Maximum temperature on the day had the highest correlation with total fatalities (*r*_s_ = −0.611). The correlation between temperature and COVID-19 cases and fatalities were negative, which indicated that at lower temperature the number of cases and fatalities increased. Among the factors of weather, snowfall and atmospheric pressure were not significantly related to COVID-19 in Japan.

### SARS-CoV-2 clade and variant distributions during the COVID-19 pandemic

**Fig. 6.**
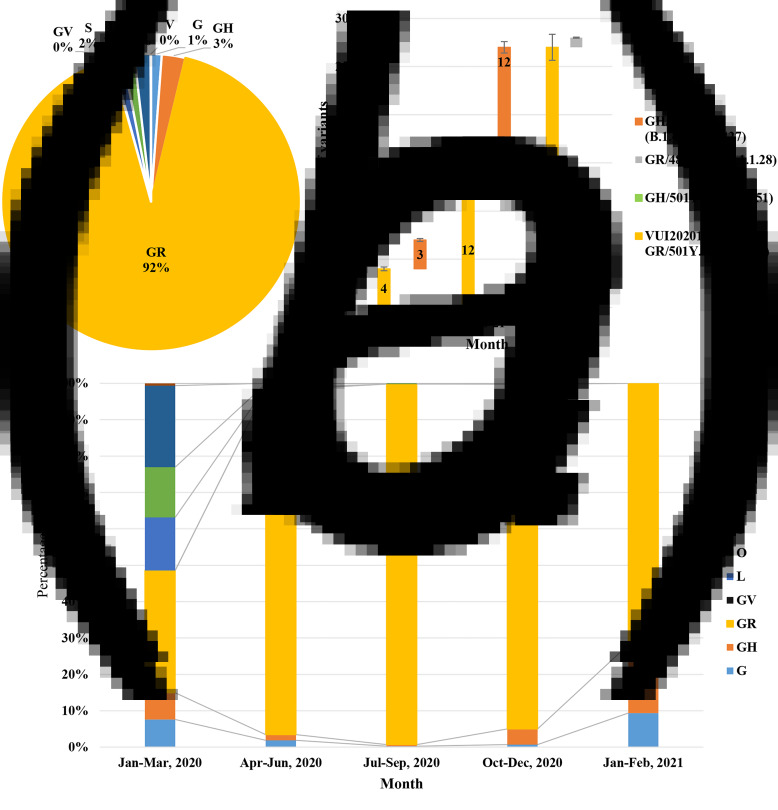
Frequency of (a) yearly clade distribution of SARS-CoV-2; (b) monthly clade distribution of SARS-CoV-2 and (c) monthly variant distributions during COVID-19 in Japan.

About 16 000 full genomes of COVID-19 were analysed from Japan. Temporal distribution of clade and variant were determined. Clade GR (92%) was the most frequent followed by GH (3%), S (2%), L (1%), O (1%) and G (1%), respectively (Fig. 6a–c). The frequency of G and GH increased during January to February 2021. The highest frequency of GR was also detected during January to February 2021 (45%) followed by October to December 2020 (35%). Variants with high transmission rate and immune escape capability were also detected in Japan during December 2020 to February 2021. Variant 501Y.V1 was prevalent (43 isolates) followed by 452R.V1 (16 isolates) (Fig. 6a–c).

### Distribution of frequency of mutations, age and genders in patients with COVID-19

**Fig. 7.**
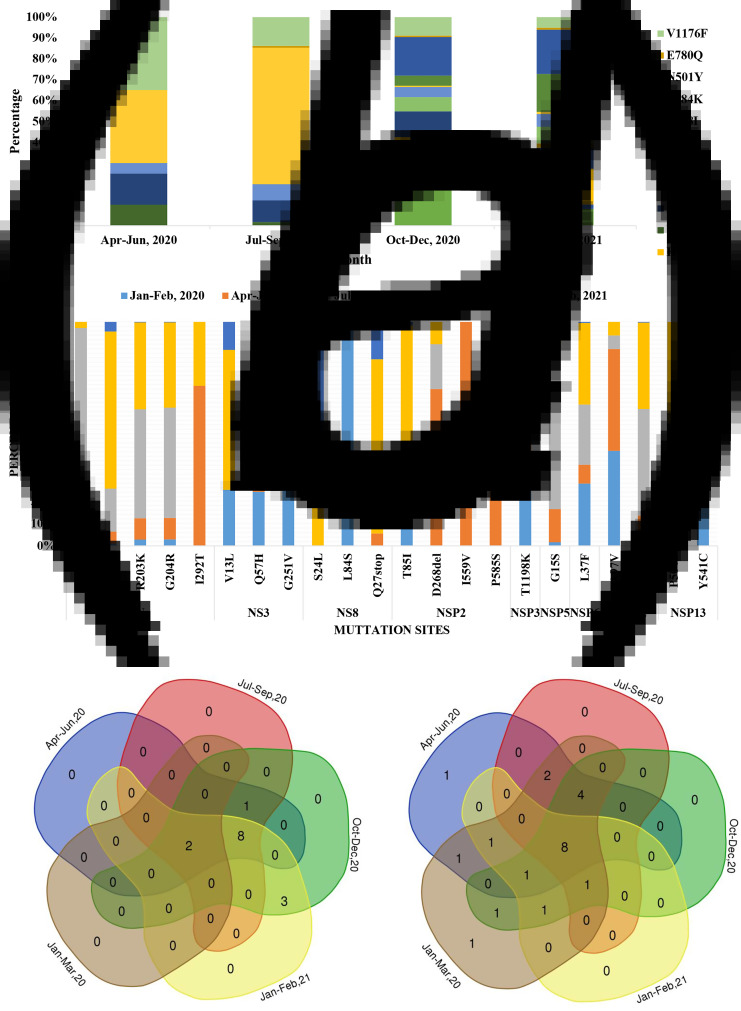
Circulation and frequency distribution of substitution and deletion mutations at (a) spike protein and (b) structural and non-structural proteins in Japan during the COVID-19 pandemic. Venn diagram depicting the point mutations at (c) spike and (d) other structural and non-structural proteins circulating throughout the pandemic in Japan.

Isolates containing various substitution points and deletion mutations were detected in Japan. About 16 000 whole genome from Japan were analysed for mutation frequency. Mutations were detected throughout the whole genome of isolates in Japan. Deletions at both the 5′ UTR and 3′ UTR were common. At spike protein, D614G was detected in about 100% isolates. Substitution mutation, T478I (55%) at spike protein were predominant during April 2020 to September 2020 (Fig. 7a–d). However, during the second wave starting from October 2020 substitution point mutations E780Q, K417N, T478I, N501Y, E484K, N439K, V1176F, S477N and A222V became common at spike protein in the isolates in Japan (Fig. 7a–d).

Deletion and substitution mutations at nucleocapsid (N) and other nsps were also frequent in Japanese isolates. Although substitution mutations, R203K, G204R at nucleocapsid and P323L at NSP12 were detected in ~90% of the isolates in Japan, the frequency reduced during January to February 2021. Among 22 point mutations at N and other eight non-structural proteins, eight namely, N_S194L, N_R203K, N_G204R, NS3_Q57H, NSP2_T85I, NSP5_G15S, NSP6_L37F and NSP12_P323L were persistent throughout the COVID-19 pandemic in Japan (Fig. 7a–d).

**Fig. 8.**
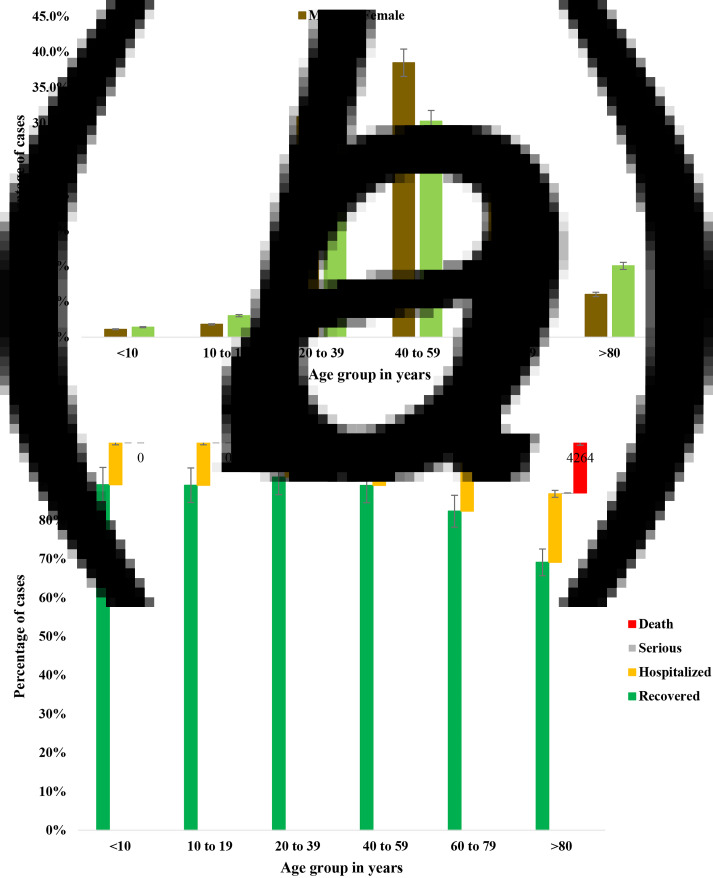
(a) Gender distribution of COVID-19 patients and (b) health outcome in COVID-19 patients in different age groups in Japan.

Both the gender and age distribution of patients with COVID-19 in Japan were analysed. The ratio of male to female patients was 1.35:1 in Japan. However, female was predominant in the age group of 20–39 years and above 80 years (Fig. 8a and b). The highest frequency of fatality was detected in patients aged above 80 years (65%), followed by the age group 60–79 years (32%) (Fig. 8a and b).

### Correlation analysis between mutation frequency and weather parameters

Novel coronavirus has acquired frequent mutations throughout the genome. The correlation between weather and mutation frequency as well as host factors and mutation frequency were determined for the isolates of Japan by Spearman's correlation analysis (Tables 4 and 5). Point mutation at RBDs namely, Y145del had strong correlation with maximum temperature (*r*_s_ = 0.61), snow fall (*r*_s_ = 0.41), N501Y with maximum temperature (*r*_s_ = 0.46) and sun hours (*r*_s_ = 0.49), N439K with minimum temperature (*r*_s_ = 0.43), A222V with sun hours (*r*_s_ = 0.51), E484K with sun hours (*r*_s_ = 0.53), T478I with RH (*r*_s_ = 0.49) and E780Q with RH (*r*_s_ = 0.43). The highest correlation between mutations of nucleocapsid protein and weather was detected for N_I292T and UV (*r*_s_ = 0.68), followed by snowfall (*r*_s_ = 0.67) and rainfall (*r*_s_ = 0.61). Significant correlation was detected between NSP5_G15S and UV (*r*_s_ = 0.91), followed by NS8_S24L and maximum temperature (*r*_s_ = 0.51), NSP3_T1198K and RH (*r*_s_ = 0.45), respectively (Table 5). Frequency of mutations and weather were positively related to each other. The impact of different host factors such as coinfection, age and gender variability on mutation frequency was also analysed. Significant correlation between host factors and frequency of mutation was detected (Tables 4 and 5).

**Table 4.** Spearman's correlation coefficients of weather factors, host factors and frequency of mutation at spike protein in Japan

Mutations	Max temp	Avg temp	Min temp	UV	Sun hours	RH	Rain fall	Snow fall	Age	Gender
L18F	0.001	0.02	0.01	–	–	0.002	–	0.01	0.005	0.04
T20N	–	–	–	0.002	0.14	0.04	0.2	0.31	0.12	0.34
H69del	0.21	0.26	0.18	0.14	0.17	0.18	0.19	0.02	–	0.15
Y145del	0.41*	0.16	0.14	0.11	0.14	0.04	0.003	0.61*	0.26	0.001
A222V	0.11	0.27	0.01	0.42	0.51	0.03	0.13	0.24	0.31	0.06
K417N	0.01	0.001	0.1	0.38	0.40**	0.41	0.02	0.31	0.46*	0.64*
N439K	0.14	0.07	0.43	0.24	0.001	0.34	0.35*	0.09	0.002	0.07
S477N	0.11	0.41	0.004	0.19	0.02	0.17	0.001	0.19	0.42	0.43*
T478I	0.01	0.18	0.12	0.42	0.41	0.56*	–	0.41	0.12	0.28
E484K	0.16*	0.19	0.014	0.11	0.53**	0.41	0.19	0.16	0.007	0.07
N501Y	0.46*	0.34	0.04	0.41	0.49*	0.001	0.43	0.39	0.19	0.09
E780Q	0.31	0.16	0.41*	0.01	0.17	0.43*	0.15	0.25	0.34	0.47*
V1176F	0.17	0.24	0.14	0.7**	0.4	0.25	–	0.24	0.01	0.13

**, * stand for 1% and 5% levels of significance.

Value between +1 and −1 are considered statistically significant.

**Table 5.** Spearman's correlation coefficients of weather factors, host factors and frequency of mutation at structural and non-structural proteins in Japan

		Environmental and host factors									
Mutation sites		Max temp	Avg temp	Min temp	UV	Sun hours	RH	Rain fall	Snow fall	Age	Gender
N	P13L	0.01	0.01	0.18	0.25	0.52*	0.01	0.17	0.08	0.023	0.004
	S194L	0.01	0.08	0.12	0.26	0.01	0.08	0.16	0.01	0.16	0.06
	R203K	0.001	0.004	0.17	0.19	0.02	0.004	0.01	0.18	0.34*	0.03
	G204R	0.01	0.06	0.01	0.23	0.03	0.06	0.03	0.21	0.28	0.31
	I292T	0.004	0.11	0.32	0.61	0.06	0.11	0.61**	0.67**	0.41*	0.1
NS3	V13L	–	0.24	0.18	0.34	–	0.24	0.13	0.31	0.51*	0.16
	Q57H	–	0.36	–	0.01	–	0.36	0.41	0.01	0.19	0.06
	G251V	0.4	0.42*	0.14	0.43	–	0.42	0.19	0.61	0.24	0.41*
NS8	S24L	0.51*	0.34	0.14	0.19	0.41*	0.34	0.13	0.14	0.61*	0.04
	L84S	0.06	0.02	0.04	0.17	0.16	0.02	0.001	0.24	0.13	0.01
	Q27stop	0.32	0.001	0.04	0.08	0.22	0.001	0.07	0.01	0.25	0.21
NSP2	T85I	0.17	0.31	0.12	0.19	0.37	0.31	0.42	0.45**	014	0.34*
	D268del	0.06	0.01	0.05	0.38	0.02	0.01	0.13	0.01	0.11	0.33
	I559V	0.09	–	–	0.34	0.19	–	0.01	0.37	0.01	0.24
NSP2	P585S	0.16	–	–	0.43	0.11	–	0.61	0.40*	0.25*	0.06
NSP3	T1198K	0.24	0.45	0.14	0.53	0.29	0.45*	0.16	0.19	0.81*	0.25
NSP5	G15S	0.05	0.4*	0.36	0.91*	0.09	0.4	0.33	0.24	0.14	0.26*
NSP6	L37F	0.25	0.34	0.16	0.24	0.24	0.34	0.19	0.29	0.015	0.16
NSP12	A97V	0.16	0.26	0.13	0.38	0.14	0.26	0.14	0.11	0.18	0.42*
	P323L	0.23	0.15	0.14	0.46*	0.21	0.15	0.18	0.61**	0.28*	0.019
NSP13	P504L	–	0.11	0.19	0.24	0.41*	0.11	0.27*	0.01	0.19	0.14
	Y541C	0.04	0.006	0.03	0.38	0.001	0.006	0.09	0.01	0.17	–

**, * stand for 1% and 5% levels of significance.

Values between +1 and −1 are considered statistically significant.

## Discussion

Evolution of new variants and increase in transmission rate of COVID-19 have infected millions of people within short time during the second wave globally [34, 35]. Numerous factors including hosts health conditions, comorbidity, age, gender and weather parameters are regulating the transmission rate and outcome of the pandemic [36–38]. Parameters of weather always play significant roles in determining the outcome and severity of infectious viral diseases. The wave of COVID-19 pandemic is also shaped by meteorological parameters namely, temperature, RH, UV index, rain fall, snow fall, atmospheric pressure and wind speed [21–28, 36–39]. Besides, direct man to man transmission is the major mode of spread of COVID-19. Gatherings of people during social, religious and sports events and exportation of new variants to specific country by immigrants are also regulating the spread of COVID-19 [21–23]. Previously different studies have found a significant impact of weather on coronaviruses infection [38–40]. High temperature and UV can reduce the survival period and viability of SARS-CoV-2 [21–24, 26, 27, 36–39]. Temperature above 40 °C can reduce the viability of SARS-CoV, MERS-CoV and SARS-CoV-2 significantly under both laboratory and environmental conditions [40–42]. In this study, the correlation and impact of various regulatory factors on the spread and outcome of COVID-19 in Japan were determined. A sharp increase in cases and fatalities associated with COVID-19 was detected after October 2020 when the temperature and UV index started to decrease significantly.

Various meteorological parameters are correlated with the COVID-19 pandemic [21, 22]. This study detected a significant correlation of weather and outcome of COVID-19 in Japan, which is similar to previous findings in Bangladesh, China, USA, Turkey and Indonesia [21–28, 36–40]. Three time frames were used to determine the correlations. We included more parameters and longer analysis for Japan compared to the previous studies. Except for atmospheric pressure, 11 other factors of weather were significantly correlated with the increase of cases, fatalities, growth rate and case fatality rate. COVID-19 incidence was strongly correlated with average temperature (*r*_s_ = −0.51), minimum temperature (*r*_s_ = −0.55), UV index (*r*_s_ = −0.42), sun hours (*r*_s_ = −0.49), RH (*r*_s_ = −0.47), total population (*r*_s_ = −0.59) and population density (*r*_s_ = −0.47). This statement is in good agreement with previous findings in Bangladesh and China [22]. Fatalities of COVID-19 was significantly correlated with population density (*r*_s_ = −0.81), total population (*r*_s_ = −0.72), RH (*r*_s_ = −0.59) and minimum temperature (*r*_s_ = −0.54). In a previous study, the association of temperature and COVID-19 fatalities was also reported from Bangladesh [22]. Furthermore, the case fatality rate and growth rate of cases were also affected by weather and population density in Japan. The findings on the impact of weather and population on COVID-19 case fatality rate and transmission rate are eccentric in this study. Sharif and Dey [22] and Şahin [36] detected a significant association of weather with COVID-19 cases in Bangladesh and Turkey, respectively. This study detected stronger correlation among weather and COVID-19 in Japan. Similar to previous studies, we also found significant correlation between UV index and COVID-19 cases and fatalities. Positive correlation was persistent between wind speed, population density, total population and COVID-19 cases and fatalities in Japan which has similarity to previous findings in Bangladesh, USA and China [22, 25, 39]. Among three time frames, the first day of incidence had the strongest correlation with the weather and total population and population density.

Other regulatory factors including duration of lockdown, availability of health facilities, availability of vaccine, tendency of the people to practice COVID-19-associated health rules, frequency of social and religious gatherings and regulation on immigrants have also shaped the wave of COVID-19 [22, 39]. Before October 2020, COVID-19 was well controlled in Japan, but with the change in weather, increase of international immigrations and circulation of new variants both the cases and fatalities increased in Japan [20].

Various substitution point mutations throughout the gnome of SARS-CoV-2 accelerated the origin of new variants [29, 32]. Among these point mutations, cluster mutations at receptor binding domains of SARS-CoV-2 are the most significant in determining the transmission capability and antibody neutralisation effect on the virus. Vaccines of COVID-19 have become available recently. However, emergence and spread of new variants have threatened the efficacy of vaccine globally. In Japan, GR (92%) was the most prevalent clade followed by GH (3%) clade. Since December 2020, four variants with high significance including 501Y.V1, 501Y.V2, 452R.V1 and 484K.V2 have been detected in Japan. These variants have been involved in high transmission rate, escape from immune reaction and lower detection rate in several countries [29, 32].

Diversity and frequency of mutations at spike protein have increased after October 2020 in Japan. About all of the isolates in Japan contained D614G at spike proteins, N_S194L and N_R203K at the nucleocapsid region. Variants with substitutions namely, K417N, T478I, N501Y, E484K, N439K and S477N at RBDs have been detected recently in Japan. Both deletion and substitution mutations at structural and non-structural proteins have become abundant in Japanese isolates. This study reported the cumulative mutation frequency in SARS-CoV-2 for the first time during the last 14 months in Japan.

We analysed the impact of weather, population and host factors on the origin and circulation of substitution point and deletion mutations in this study. A previous study in Bangladesh has reported significant correlation between average temperature and mutation frequency at ORF1ab and at S- D614G [22]. Several research studies on influenza virus have reported the impact of weather and UV radiation on the evolution of substitution mutations [41, 42]. Similar to previous studies, we also detected notable correlation between weather and mutation frequency of Japanese isolates, and between host factors and mutations [21–28, 36–40]. Weather factors including temperature, UV, RH, snow fall, rain fall and sun hours were significantly correlated with the frequency of mutations at receptor binding domains of spike proteins and other structural and non-structural proteins. Furthermore, age and gender of patients were also correlated with the frequency of important mutations. However, unlike previous studies in Bangladesh, we did not detect any significant correlation between comorbidity and mutation frequency in Japan [22].

In the demographic analysis, this study found that the male to female ratio of the infected patients was not significantly different. The outcome of COVID-19 was severe in patients aged above 60 years. Both death rate and hospitalisation rate were higher in patients aged above 60 years in Japan. The findings on both the distribution of age and gender of COVID-19 cases in Japan were similar to previous studies [19].

The impact of weather on the increase of cases and fatalities in Japan was evaluated in this study. This study has reported the association of weather and host factors with the circulation of isolates with mutations for the first time in Japan. A complete analysis on the present variants and clades of the circulating SARS-CoV-2 in Japan during the last 14 months was conducted in this study. This study will help to take proper implications by providing the crucial information on the impact of weather, population and host factors on the frequency of mutations and outcome of COVID-19. In future, studies including more data on mutation of isolates and on clinical outcomes can be conducted based on this research to create a complete scenario of the COVID-19 pandemic. This study will work as a significant resource of information for future studies on environmental correlation with the COVID-19 pandemic. Crucial information on circulating variants and mutations in this study will work as a guideline for future studies focusing on the evolutionary dynamics of COVID-19.

## Conclusion

This is one of the early studies focusing on the impact of weather on the COVID-19 pandemic in Japan. This study has created a complete concept on the seasonal pattern of the cases and fatalities of COVID-19 in Japan during the last 14 months. This study included numerous factors that can affect the outcome of the pandemic to understand the dynamic of COVID-19. Significant correlation was detected between weather and cases, weather and fatalities, population and cases, population and fatalities, weather and mutations, host factors and mutations. Four recently evolved variants including 501Y.V1, 501Y.V2, 452R.V1 and 484K.V2 have been reported in Japan. This study concludes that not only preventive measures and interventions, but also weather has significant roles in shaping the outcome and severity of the COVID-19 pandemic. This study will help international health organisations and policy makers to understand the COVID-19 pandemic for taking appropriate steps to minimise the disease burden.

## Data Availability

Restrictions apply to the availability to the data that support the findings of this study.
